# Microbiological and Enzymatic Activity Modulates the Bitter Taste Reduction in Decanted Coratina Olive Oil

**DOI:** 10.3390/foods11060867

**Published:** 2022-03-18

**Authors:** Gino Ciafardini, Biagi Angelo Zullo

**Affiliations:** Department of Agricultural, Environmental and Food Sciences, University of Molise, Via De Sanctis, 86100 Campobasso, Italy; ciafardi@unimol.it

**Keywords:** bitter taste, Coratina, extra virgin olive oil, oleuropein, olive oil decantation

## Abstract

Coratina monocultivar extra virgin olive oil (EVOO) is known for its level of bitterness, which, if too high, can cause consumer acceptance problems. The aim of this study was to modulate the bitter taste of freshly produced olive oil through endogenous enzymatic activity and microbiota during the decantation phase. The opalescent appearance of the newly produced EVOO was substantially reduced during the first three months of decantation due to the deposition of more than 90% of suspended material, consisting of vegetation water and suspended solid particles. The high content of biophenols and the reduction in water concentration in the oil samples negatively affected the survival of yeasts, which were absent in the oil samples at the end of the third month of decantation. The oleuropeinolytic activity was very intense during the first month of decantation, whereas the reduction in the bitter taste associated with the aglycons was consistent only in the second and third months of decantation. At the end of decantation, the sensory notes of bitterness in the Coratina EVOO were reduced by 33%, lowering the position on the value scale without altering the other qualitative parameters whose values fell within the limits of the commercial EVOO class.

## 1. Introduction

Extra virgin olive oil (EVOO) is a vegetable oil extracted from fresh and healthy olives (*Olea europea* L.) by mechanical processes and is highly appreciated worldwide for its flavor and beneficial effects on human health [1]. EVOO is the highest grade olive oil that can be consumed directly without undergoing further refining processes. The health benefits of olive oil have been officially recognized by the European Food Safety Authority (EFSA) [2]. Fresh-squeezed EVOO is characterized by opalescence due to the presence of micro-droplets of vegetation water and solid particles, with an intense and robust flavor that can be more or less accentuated depending on the total polar phenols it contains. Polar phenols play a fundamental role in determining the sensorial profile, and each EVOO is unique to each variety and year of production. The sensorial characterization of olive oil includes both positive and negative taste attributes. Positive taste notes included fruitiness, pungency, and bitterness. Bitterness and pungency sensations are considered to be two of the most important parameters used to identify good-quality EVOO, and when these sensations are perfectly balanced with each other and accompanied by pleasant fruitiness notes, an EVOO is considered to be of excellent quality. Taste plays a significant role in human food selection. Bitter taste perception involves the detection of numerous structurally divergent bitter compounds [3]. Many natural substances produce bitterness and elicit aversive responses in humans, suggesting that bitterness transduction is a key defense mechanism against harmful substances [4]. Coratina is an Italian olive variety that produces EVOO with a medium-high phenol content, renowned for its strong pungency and bitterness sensation used to raise the phenolic content of other EVOOs through blending. However, although this parameter represents an important positive sensory attribute; excessive bitterness can be perceived negatively, and in some cases, consumers can reject completely bitter olive oils [5,6]. The phenolic content of olive oil depends on the olive variety; however, climatic conditions, olive maturity, and processing may also affect the amount of total phenols in the oil [7]. Several physical treatments, such as cold storage or heating of olives, have been proposed to reduce the excessive bitterness of olive oil [8,9]. Another study demonstrated that liquid–liquid extraction using water as a solvent was a viable method for reducing the concentration of phenols and the bitterness of Arbequina extra virgin olive oil [10]. However, the strong bitterness and pungency taste of freshly produced Coratina EVOO can be attenuated during the storage of the product because of the processes of hydrolysis, oxidation, auto-oxidation, and polymerization promoted by the enzymes coming from the fruit and the oil-borne microbiota composed mainly of oleuropeinolytic yeast species [11,12,13]. Georgalaki et al. [14] demonstrated the presence of hydrolytic and oxidative enzymes that may reduce the pungency and bitterness sensory notes in EVOO, strictly linked to the content of secoiridoids, including oleuropein, ligstroside, and dimethyl oleuropein, as well as their phenolic derivative products. Ciafardini and Zullo [15] demonstrated that yeast activity in EVOO can positively affect the sensory properties of the product through the production of hydrolytic enzymes, such as β-glucosidase and esterase. In fact, in a previous study, during the first three months of storage, researchers were able to reduce the bitter taste of an oil rich in phenols produced by the Don Carlo variety through blending with newly produced Leccino EVOOs, which can provide high oleuropeinolytic activity [16]. However, this system is advantageous for the production of EVOOs consisting of blends of oils extracted from two or more varieties but not for the production of Coratina monocultivar EVOO. In the past, producers used to carry out the harvest when the Coratina fruits were completely ripe, and the oil they produced presented an advanced state of oxidation and therefore tended to give no or only a slight bitterness sensation. Today, however, Coratina monocultivar EVOO, which has a highly bitter taste, can be consumed directly after a racking operation, which generally reduces the bitter character of oil due to the enzymatic hydrolysis of secoiridoids. Racking is performed by storing freshly produced EVOO in tanks, allowing it to settle for 5–6 months, during which time the oxidizing and oleuropeinolytic enzymes (β-glucosidase and esterase) degrade some of the phenolic compounds in the oil fraction, improving its sensory characteristics. However, if this system is not conducted adequately, it has drawbacks that discourage its use. In fact, the storage of the oil for long periods in contact with the sludge before being packaged for sale constitutes a potential problem for the industry, which risks altering the sensory characteristics of EVOO due to the activity of microbiota that settle within suspended solids and vegetation water in the sludge [15,17]. As an alternative, in some cases, this problem can be solved by resorting to filtration, which removes water and solid particles with enzymes and microbiota. Given the scarcity of scientific indications for the solution to the aforementioned problem, the purpose of this work was to develop a strategy for the biological reduction of the excessively bitter taste of Coratina monocultivar EVOO through the enzymatic hydrolysis of the phenolic compounds of the oily mass. To this end, EVOO samples were subjected to decantation, after which racking was accomplished before the final storage of the product.

## 2. Materials and Methods

### 2.1. Production of Coratina Monocultivar EVOO

Olives from a 50-year-old orchard located in the province of Bari (Apulia region in southern Italy) harvested during the 2020 harvest year were used in this study. A homogeneous mass of healthy olives harvested at the beginning of ripening, after being freed from the leaves and other foreign materials, was washed and subjected to EVOO extraction within 12 h of collection. The oil was extracted using a three-phase FR 350 mini-crusher (Mori-TEM s.r.l., Tavernelle, Florence, Italy). The fruits were first ground with a grinder at 2000 rpm, and the resulting oily paste was subjected to malaxation for 20 min at 27 °C. Prior to oil extraction, the paste was moistened with tap water and then subjected to double separation by horizontal (decanter) and vertical centrifugation. Freshly produced Coratina EVOO was placed in a stainless steel tank and immediately used in the decantation tests.

### 2.2. Decantation Trials

A mass of freshly produced Coratina monocultivar EVOO was stored under a nitrogen atmosphere in three 200 L stainless steel tanks with a conical bottom equipped with a mud drain valve, within which the decantation process took place for a period of three months. At the beginning of the experiment and during each month of decantation from each container, 12 L of oil was taken with a silicone tube connected to a suction pump, of which 2 L was subjected directly to the various laboratory analyses, while the remaining 10 L was transferred to an empty metal tank and stored for a maximum period of seven months at 12–14 °C. The oil samples, taken every month at a depth of 10 cm from the oil surface of each container, made it possible ensured three repetitions for each treatment. At the same time, to prevent the onset of sensory defects during the three months of decanting, at each sampling, the sludge collected on the conical bottom of the containers was eliminated through the drain cock, while the air in the headspace of each container was replaced with a stream of nitrogen introduced above.

### 2.3. Solid Particle and Water Contents

The solid particle and water content of Coratina monocultivar EVOO were evaluated each month during the three months of decantation and at the end of storage using the methods described by Zullo et al. [18]. Briefly, the solid particle content was assessed using 30 g of olive oil sample. The sample was filtered under reduced pressure through a 0.45 µm pre-weighed and oil-wetted nitrocellulose filter (Minisart NMLSartorius, Göttingen, Germany). The water content of the olive oil samples was assessed using the 37858 HYDRANAL—Moisture Test Kit (Sigma–Aldrich, Milan, Italy) following the instructions given by the manufacturer. Each analysis was repeated three times.

### 2.4. Biophenol Content

The official International Olive Oil Council (IOC) method [19] was used for the extraction, identification, and determination of the phenolic compounds. High Performance Liquid Chromatography (HPLC) analysis was performed on an Agilent 1200 liquid chromatographic series system equipped with a diode array UV-VIS detector Agilent Technologies, Santa Clara, CA, USA). A C18 column (4.6 mm i.d. × 250 mm; particle size, 5 µm) (Phenomenex, Torrance, CA, USA) coupled to a security guard C18 4 × 3.0 mm (Phenomenex) was used. Elution was performed at a flow rate of 1.0 mL min^−1^ using a mixture of water/acetic acid (97:3, *v*/*v*) (solvent A) and methanol/acetonitrile (50:50, *v*/*v*) (solvent B) as the mobile phase. The solvent gradient was changed according to the following conditions: 95% (A) −5% (B) to 70% (A) −30% (B) in 25 min; 65% (A) −35% (B) in 10 min; 60% (A) −40% (B) in 5 min; 30% (A) −70% (B) in 10 min; and 100% (B) in 5 min, followed by 5 min of maintenance. The analysis was repeated in triplicate for each olive oil sample. The contents of the total phenolic compounds and other relevant groups were quantified in mg tyrosol/Kg oil.

### 2.5. Endogenous Oleuropeinolytic Activity of Coratina Monocultivar EVOO

Among the endogenous enzymes involved in the debittering process of Coratina EVOO, the activities of β-glucosidase and esterase were evaluated.

#### 2.5.1. β-Glucosidase Activity

The crude enzymatic extract used to evaluate β-glucosidase activity was prepared as reported by Zullo et al. [16]. The crude enzymatic extract from 30 mL of oil was divided into two 6 mL fractions. One was enriched with 0.4% (*w/v*) synthetic substrate β-D-glucopyranoside (Sigma–Aldrich, Milan, Italy), whereas the sample without the substrate was used as a control. After a 20 min incubation at 30 °C, both samples were centrifuged at × *g* for 5 min. The resulting supernatants were microfiltered through Millex syringe filters with a pore size of 0.22 µm (Merck Millipore Ltd., Tullagreen, Ireland) and analyzed at 410 nm using a spectrophotometer (Model 6300; Jenway, Essex, UK). The amount of *p*-nitrophenol (*p*-NP) released was quantified to obtain a standard curve. Enzymatic analysis of each sample was repeated three times. The enzymatic results are expressed as µg *p*-NP mL^−1^ oil.

#### 2.5.2. Esterase Activity

The crude enzymatic extract used for the evaluation of esterase activity was prepared as described in Section 2.5.1, with the only difference being that the phosphate buffer (0.1 M) at pH 7 was replaced with Tris-HCl buffer (0.1 M) at pH 7.5. Esterase activity was evaluated using a spectrophotometric method based on the initial rate of increase in the absorbance at 410 nm. The esterase activity was assayed using *p*-nitrophenyl-acetate (*p*-NPA, Sigma-Aldrich, Milan, Italy) according to the method described by Romeo et al. [20], with some modifications. At the time of the assay, 200 µL of crude extract was transferred into a spectrophotometer cuvette containing 2.9 mL of Tris-HCl buffer 0.1 M pH 7.5 and 200 µL of 0.15 M *p*-NPA in ethanol. Absorbance (ABS) was recorded after 4 min of reaction at 410 nm, and the values were compared with a calibration curve prepared with increasing quantities of *p*-NP. The enzymatic results are expressed as µg *p*-NP mL^−1^ oil.

### 2.6. Microbiological Analysis

Microbiological analysis was conducted on the EVOO samples during the decantation period and after seven months of storage using the method described by Zullo and Ciafardini [21]. Briefly, 30 mL of oil sample were micro-filtered through a 0.45 µm sterile nitrocellulose filter. The nitrocellulose filter used to capture each sample was then transferred into a 25-mL sterile beaker and homogenized using a Turrax model T25 homogenizer (IKA, Milan, Italy) in the presence of sterile physiological solution. The solution was then subjected to 10-fold serial dilution. The total bacteria were determined using plate count agar standard (PCAS) medium (Oxoid, Basingstoke, Hampshire, UK) after incubation at 28 °C for three days. The total molds were evaluated in oxytetracycline glucose yeast extract agar (Oxoid) supplemented with 100 µg mL^−1^ gentamicin and 100 µg mL^−1^ chloramphenicol. The colony forming units (CFU) that developed on medium agar were counted after seven days of incubation at 28 °C. Finally, total yeasts were evaluated in malt yeast glucose peptone agar (MYGPA) medium. The MYGPA medium was supplemented with tetracycline (20 mg L^−1^) to inhibit bacterial growth. The CFU count was determined after five days of incubation at 30 °C. The yeast colonies isolated from the oil samples at racking time were then transferred to MYGPA medium plates (master plates) [22]. The master plates were set up in triplicate for the enzymatic assays and chromogenic groups.

### 2.7. Enzymatic Assays in the Yeast

Enzymatic assays in yeast involve β-glucosidase and esterase activities. Enzymatic analysis was performed using master plates containing 50 yeast colonies isolated from the EVOO samples at the racking time. All enzymatic tests were performed in triplicate. The β-glucosidase assay was performed using MYGPA medium enriched with esculin (Sigma-Aldrich, Milan, Italy) and FeCl_3_ (Sigma-Aldrich, Milan, Italy) as described by Arévalo et al. [23]. Esterase activity was evaluated as reported by Zullo et al. [18], using MYGPA medium supplemented with NaCl, CaCl_2_, and Tween 80.

### 2.8. Laboratory Inoculation of Coratina Monocultivar EVOO with Five Oil-Borne Yeast Species

Laboratory tests were performed by inoculating pure cultures of oil-borne yeast species into Coratina EVOO enriched with increasing doses of water. The yeasts used in the laboratory tests belonged to the same species found in Coratina EVOO subjected to decantation tests, which were represented by five oil-borne strains: *Barnettozyma californica* 2084, *Candida adriatica* 2087, *Kuraishia capsulata* 2090, *Nakazawaea molendinolei* 2096, and *Yamadazyma terventina* 2092. These yeasts were previously isolated in our laboratory from different EVOOs of Italian origin and were characterized by molecular analysis [18,21]. The studies conducted on the survival of the five species of yeasts were performed using Coratina EVOO samples taken from each container after three months of decantation whose physicochemical and microbiological analyses showed that the concentrations of water and biophenols were 0.07% (*w*/*v*) and 750 mg TE Kg^−1^ oil, respectively, with the absence of viable yeast cells. To this end, 1.5 L of Coratina EVOO obtained from each container with oil subjected to decantation was divided into three fractions of 0.5 L. The first was used as is, while the other two were enriched with sterile distilled water until a concentration of 0.21% (*v*/*v*) and 0.30% (*v*/*v*), respectively. Immediately afterwards, all of the oil samples were inoculated with 100 mg of microbial biomass containing the five yeast strains mixed at same ratio. The inoculated samples were vortexed for 1 min and stored in the dark for three months at 15–17 °C. Microbiological analyses were performed at the beginning of the experiment. After each month of incubation, the samples were shaken before analyzing following the method described in Section 2.6.

### 2.9. Evaluation of Predominant Yeast Species in Coratina EVOO

The yeasts isolated from the microbiological analyses performed using Coratina EVOO samples during decantation and those isolated from the inoculation test performed in the laboratory were identified by screening a large number of colonies grown on a specific chromogenic medium, as reported by Zullo and Ciafardini [21]. Approximately 1000 yeast colonies from the master plates obtained after the above microbiological analysis were transferred into the CHROMagar Candida medium (BBL, cod. 4354093; Heidelberg, Germany) and assayed after seven days of incubation at 30 °C. All of the yeast colonies inoculated on the chromogenic medium based on the cell morphology, color of the colonies, and formation of pseudohyphae were grouped into homogeneous chromogenic groups as follows: (1) uniform white; (2) uniform bordeaux; (3) mucous white; (4) smooth violet cream; and (5) uniform bluish. From each chromogenic yeast colony group, 10 isolates were randomly chosen and used for identification at the species level by sequencing the D1/D2 region (approximately 600 bp) of the large (26S) ribosomal subunit gene using NL1 and NL4 primers, following the protocols described by Kurtzman and Robnett [24].

### 2.10. Sensory Notes, Bitterness Index (K_225_), and R-Index Analysis

The sensory and bitterness index analyses of Coratina monocultivar EVOO were performed using olive oil samples both collected during the decantation period and after seven months of storage. Sensory analyses were performed by a fully trained analytical taste panel recognized by the Italian Ministry of Agriculture. The panel test was conducted using the IOC standard profile sheet method [25]. Each taster analyzed the samples during three different sessions. The median values of the sensory data were calculated according to the IOC method [26]. In addition to the sensory analysis mentioned above and reported in the results, a quantitative descriptive analysis of the sensory attributes of bitterness was performed by the same panel using a structured five-point scale: 0 indicates the absence of an attribute, 1 simple perception, 2 light presence, 3 middle presence, 4 strong intensity, and 5 the highest intensity [8]. The bitterness index (K_225_) was evaluated following the method described by Beltrán et al. [26], with some modifications. One gram of oil was transferred to a 10 mL screw-capped Pyrex tube containing 1 mL of a mixture of methanol and water (80:20, *v*/*v*). The heterogeneous mixture was vortexed for 3 min and centrifuged at 5000× *g* for 5 min. The overlying methanolic phase was recovered and transferred to another 10 mL Pyrex tube. Thereafter, extraction was repeated two more times. All phenolic extract masses obtained were first microfiltered through Millex syringe filters with a pore size of 0.22 µm (Merck Millipore Ltd., Tullagreen, Ireland) and then diluted to a final volume of 100 mL using the same mixture used for extraction. The absorbance of the phenolic extract was measured at 225 nm against a methanol: water (80:20, *v*/*v*) mixture in a quartz cuvette. The analysis of each sample was repeated three times. The R-Index was determined as follows [27]:

R-Index = (T + HT)/(T + HT + OL_der_ + LIG_der_)

where the numerator shows the sum of the tyrosol (T) and hydroxytyrosol (HT) contents, and the denominator shows the sum of the (T), (HT), derivative oleuropein (OL_der_), and derivative ligstroside (LIG_der_) contents.

### 2.11. Analytical Indices

Both the oil samples taken during the decantation period and those from the masses of decanted oil stored for seven months were subjected to conventional chemical analysis to establish the commercial merceological class. The free fatty acid content, peroxide values, and UV spectrophotometric indices (K_232_, K_270_, and ∆K) were measured in triplicate for each sample according to European Community Regulation 1348/2013 [28].

### 2.12. Statistical Analysis

Statistical software (ver. 7.0) was used for data processing (StatSoft for Windows; Tulsa, OK, USA). Means were compared using Duncan’s multiple-range test (one-way ANOVA). Differences were considered statistically significant at *p* < 0.05.

## 3. Results and Discussion

EVOOs characterized by a high intensity of bitter notes can be rejected by consumers in markets accustomed to the milder taste of seed or refined oils, obtained by solvent extraction. The Coratina cultivar is an Italian ancestral variety characterized by a bitter taste, which in many cases is excessively high, depending on the time of fruit harvesting and the geographical area of production. This does not imply automatic rejection of the oil because freshly produced olive oils are normally bitter; however, an excessively high level of bitterness can cause problems in terms of consumer acceptance [5]. While it is often necessary to delay the fruit-harvesting period to obtain a Coratina EVOO with an adequate level of bitterness, the extension of the duration of the drupes remaining on the tree can cause spontaneous fruit fall and a deterioration of the physicochemical quality of the oil produced. In terms of technology, the excessively bitter taste of Coratina EVOO can be modulated by first subjecting the oily mass to decantation, followed by the racking of the oily mass before storage or packaging.

### 3.1. Effects of Decantation Time on the Physicochemical Characteristics of Coratina EVOO

Freshly produced Coratina EVOO has a high content of suspended solids, colloids, and micro-drops of vegetation water, which are associated with the microbiota that make up the biotic fraction of the oil. During the decantation of the product, some of the suspended materials (solid particles and vegetation water) and microorganisms move to the bottom of containers, creating a water-rich habitat favorable for the growth of the harmful yeast species responsible for sensory defects in the final product [29]. Solid particles, including microorganisms that are often entrapped in micro-drops of vegetation water suspended in olive oil or micro-drops, are adsorbed on the solid particle surface, creating a water film [15,17]. The size of suspended particles depends on the water content, endogenous amphiphilic molecules, extraction procedure, and storage time [17,30]. The water content in fresh olive oil taken at the decanter has been reported to range between 0.10% and 0.80%, with micro-drop sizes between 1 and 20 µm [17,18,30,31,32]. The role of water in EVOO stability has been largely discussed in the literature, whose presence can be positively or negatively associated with oxidation, hydrolysis, and microbial activity. The International Olive Oil Council (IOC) states that, to ensure durability, EVOO must have less than 0.20% water content [32]. In this study, the contents of solid particles, vegetation water, and phenolic compounds decreased in the extracted oils according to the decantation time (Table 1). The suspended materials of the EVOO consist of solid particles and micro-drops of vegetation water in freshly produced oil with a typical veiled appearance. In three months, the oily mass was depleted of suspended materials owing to the decantation process. The solid particle concentration decreased from 0.140% to 0.012% (*w*/*v*) after three months of decantation (Table 1). Freshly produced Coratina EVOO had a water content equal to 0.21% (*w*/*w*); however, the micro-droplets of vegetation water settled quickly during the first two months of sedimentation, reducing the water content in the oily mass from 0.21% to 0.08% and 0.07% after two and three months of sedimentation, respectively. In the first two months of decantation, the reduction in water content reached 61%, while in the third month it rose to 67%. The opalescent aspect of the newly produced Coratina EVOO disappeared after three months of decantation, during which more than 90% of the suspended materials comprised of solid particles and water settled at the bottom of the containers (Table 1). This behavior is in agreement with the results of previous tests performed on short-lived veiled EVOO upon storage in the dark [11]. The concentration of the total polar phenols recorded during the decantation time decreased from 773 mg TE kg^−1^ oil in the freshly produced Coratina EVOO to 758, 753, and 750 mg TE kg^−1^ at the end of the first, second, and third months, respectively. However, the greatest reduction in the phenolic content (2%) compared to the control was recorded in the first month of decantation (Table 1). This result can be related to the high decay of the solid materials and the water content recorded in the first and second months of decantation. In fact, it is known that, in addition to the aqueous fraction, the solid particles are rich in phenolic compounds that are removed from the oily mass because of the decantation process [11,17,33,34].

### 3.2. Oleuropeinolytic Activity of EVOO during Decantation

The endogenous enzymatic activity of Coratina EVOO, attributed to the oleuropeinolytic enzymes β-glucosidase and esterase, was higher in the freshly produced olive oil, where it remained almost unchanged during the first month of decantation; by contrast, it was significantly lower in the following months. Compared to the freshly produced oil (control), the β-glucosidase activity at the end of the second and third months of decantation recorded a decay of 45% and 55%, respectively. Esterase activity also decreased by more than 50% after the first month of decantation (Table 2).

However, if we consider that, in general, endogenous enzymes can be derived from both fruit fragments [35] and from the oil microbiota [15], the greater enzymatic activity recorded in freshly produced olive oil samples and in those racked during the first month of decantation can be linked to their higher content of suspended solids and vegetation water, which, in addition to favoring microbial activity, both represent an important source of enzymes that remain active for a long time, including the storage period of the product (Table 1 and Table 2).

### 3.3. Microbiological Analysis and Survival of Yeasts in the EVOO of Coratina

Freshly produced Coratina EVOO contained yeasts and bacteria, while fungi were absent. However, during decantation, the bacteria disappeared in all oil samples, while the yeasts gradually reduced in the first two months until they disappeared completely at the end of the third month of decantation. Finally, microbiological analyses carried out after seven months of storage showed the presence of yeasts only in the freshly produced oil samples (Table 3). The percentage of β-glucosidase^+^ yeast strains remained almost unchanged during decantation, whereas those producing esterase decreased in samples subjected to more than one month of decantation (Table 3).

The species of oil-borne yeasts that characterize the microbiota of the oil varied during the decanting process. In fact, in the samples of freshly produced olive oil, the presence of the yeast species *Barnettozyma californica*, *Candida adriatica*, *Kuraishia capsulata*, *Nakazawaea molendinolei*, and *Yamadazyma terventina* was observed. During decantation, however, some species disappeared, and only *C. adriatica* and *N. molendinolei* remained viable in the oil (Table 4).

**Figure 1:**
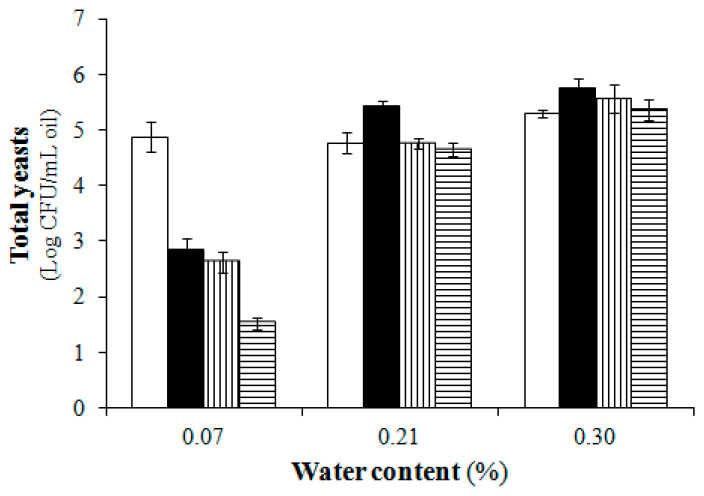
Survival of yeasts in Coratina monocultivar EVOO with different water contents during incubation. Time zero (□); one month (■); two months (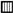); three months (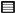).

As shown in Table 3 and Table 4, the survival of yeasts in the EVOO of Coratina during decantation did not exceed two months. These results, in agreement with previous findings [11,15], seem to depend on the high phenolic content of EVOO from Coratina and on the greater intensity of decantation of the suspended material, rich in yeasts, in the first two months of decantation (Table 1). However, the results in Table 1 also point to a strong reduction in the water content of the oil samples, which represents another parameter capable of strongly influencing the survival and microbial activity in the oil [36]. The hypothesis that viable yeast cells in the EVOO of Coratina were penalized not only by the high phenolic concentration but also by the low residual water content in the decanted oil was confirmed by the direct inoculation test of the oil, performed separately in the laboratory, using the same oil and the same yeast species. From the results of this second test shown in Figure 1, it can be seen that, with the same phenolic concentration, the water content of 0.07% strongly decreases the survival of the yeasts, despite the high initial concentration of yeasts in each milliliter of oil.

Finally, it is interesting to note that, also in this test, the two yeast species known as *C. adriatica* and *N. molendinolei* showed, unlike the other species inoculated in the EVOO of Coratina, the best performance in terms of survival (Table 4), thus confirming the results reported in Table 5.

### 3.4. Secoiridoid Hydrolysis and Bitterness Decay in Coratina EVOO during Decantation

The reduction in the bitter taste of freshly produced oil is largely due to the enzymatic hydrolysis of secoiridoids (oleuropein, ligstroside, and their derivatives). Enzymatic activity in virgin olive oils was reported by Georgalaki et al. [14], whereas the presence of proteins was first reported by Hidalgo et al. [37] and further verified by other authors [35,38]. These enzymes, which originate mainly from seeds, are released from the fruit to the oil during the extraction process. However, Ciafardini and Zullo [15] demonstrated for the first time that the oleuropein present in EVOOs can be hydrolyzed by β-glucosidase produced by some yeasts, which represent the biotic fraction of freshly produced olive oil. The enzymes from the fruit together with those from the microbiota contribute to the positive or negative physicochemical and sensorial structures of freshly produced olive oil. The oil enzymes β-glucosidase and esterase are involved in the debittering process, while other enzymes, such as lipases, can negatively affect the quality of the oil through the appearance of defects. The hydrolytic process of the secoiridoids, evaluated with the R-index, was higher in the freshly produced oil and in the one analyzed after the first month of decantation (Table 6). On the other hand, in the subsequent months of decantation, the R-index was significantly reduced with a lower production of hydroxytyrosol and tyrosol released by the hydrolysis of the secoiridoid derivatives (data not shown). Consequently, in agreement with Siliani et al. [39], who demonstrated that the bitter taste of oleuropein aglycon (3,4-DHPEA-EA) is higher than that of oleuropein itself, the appreciable decay in the bitter taste of the oil evaluated by sensory tests and the bitterness index was observed only in oil samples subjected to decantation for a period exceeding one month (Table 6). The reduction in the median of the values inherent in the sensory test was highly correlated (r^2^ = 0.96) with the decantation time. The values relating to the bitterness index (K_225_) also showed the same behavior as the sensory test (r^2^ = 0.90). The reduction of the bitter taste was also highlighted during the storage of the decanted product, with appreciable results only for the freshly produced olive oil and in the one subjected to one month of decantation (Table 6). The greater hydrolytic activity (R-index) observed in freshly produced olive oil and in that subjected to one month of decantation can be attributed to their greater endogenous oleuropeinolytic activity and to the high microbial content (Table 2 and Table 3), which are both positively correlated with the higher concentration of suspended solids and vegetation water as reported in Table 1.

The sensory profile referred to other qualitative parameters evaluated during decantation and at the end of seven months of storage, indicating a reduction of 24% in the median fruitiness values in the samples submitted at one and two months of decantation with respect to the control, while the pungency attribute did not undergo significant changes. However, since the defects were zero in all samples and the median fruitiness was greater than 1, all samples were placed in the EVOO category (Table 7).

The results regarding the analytical indices highlighted the increase in the peroxide value and K_232_ in the oil samples subjected to both decantation and storage, whereas the other parameters did not undergo significant changes, albeit with small differences. However, the analytical parameter values of all oil samples analyzed fell well within the limits of the EVOO class (Table 8).

The results of the physicochemical, enzymatic, and microbiological analyses indicated the possibility of modulating the excessive bitter taste of Coratina EVOO by reducing the sensory note by 33% after three months of decantation, moving it from the “strong intensity” position (4.8) to “middle presence” position (3.2) without worsening the other quality parameters of the product during storage (Table 6). The physicochemical parameters listed in Table 1 are directly involved in the biochemical processes occurring in the oil immediately after extraction. In general, the high content of suspended solids and vegetation water promotes the survival and enzymatic activity of yeasts in oil. In contrast, the high phenolic content performs an action opposite to that of water. The dynamic balance between these parameters determines the enzymatic activities capable of improving the chemical and sensory characteristics of freshly produced olive oil during storage. One of the possible negative effects of a high water content and low phenolic concentration is to promote the growth of yeast producing lipase active on triacylglycerol. Previously, studies performed using oils endowed with phenolic compounds have found that by increasing the water content from 0.06% to 0.31%, only 33% of the oil yeasts showed lipolytic activity, whereas the excess content of water (1.31%) stimulated the production of lipase in 90% of the tested yeasts. With reference to the phenolic content, on the other hand, a reduction in lipase-producer yeasts from 100% to 11% was reported when the phenolic concentration rose from 84 to 510 mg gallic acid equivalent per kg oil. Based on these findings, it can be assumed that among the various Coratina EVOO samples analyzed in this study, the freshly produced EVOO represents the one most exposed to alterations during decantation and storage, as it is the richest in water and suspended solids (Table 1). Surprisingly, however, similar to all other oil samples, these oil samples also fell into the EVOO class at the end of storage (Table 7 and Table 8). This result could depend on the high phenolic content, which inhibits many enzymatic activities in the oil. This last aspect favors the reduction of the bitter taste in Coratina EVOO through the decanting technique, since very bitter oils are also rich in phenolic compounds. However, it should be emphasized that to safeguard the quality of the oil during decantation, it is necessary to separate the mass of decanted oil from the sludge deposited at the bottom of the containers because some unwanted biochemical reactions promoted by the microbiota, such as those of lipases, take place at the interface between the aqueous phase of the deposit and that of the oily mass. In fact, the absence of sensory defects in the Coratina EVOO samples at the end of storage could in part be explained by the fact that, with the exception of freshly produced oil, the sludge was removed from the oily mass regularly after each month. However, in some cases, it may also be necessary to filter the decanted oil before it is subjected to storage or packaging.

## 4. Conclusions

The enzymatic modulation of bitter taste, as studied in this work, offers new commercial possibilities for Coratina monocultivar EVOO. The bitter and phenol-rich Coratina monocultivar EVOO, normally used to increase the phenolic concentration of other oils, can be improved to a palatable level and marked for direct consumption after a short period of decantation that favors the partial enzymatic hydrolysis of secoiridoids and their derivatives. In the present study, the activity of oleuropeinolytic enzymes was found to be very intense during the first month of decantation, producing an increase in oleuropein and ligstroside aglycons, which are powerful bitter tastants of olive oil. The enzymatic hydrolysis of the aglycons recorded after the second and third months of decantation led to an appreciable reduction in the bitter taste. The extensively bitter and phenol-rich Coratina EVOO subjected to a decantation period of three months and the subsequent storage of the settled oil did not develop any negative sensory attributes, remaining in the EVOO merceological class. The good qualitative stability demonstrated by Coratina EVOO subjected to decantation and storage for a period of seven months can be attributed to the high phenolic concentration and rapid reduction of the water content in the first months of decantation, which inhibited microbial activity. However, considering that, in the newly produced olive oil, the yeasts capable of fast decantation as suspended materials seem to be more responsible for the defect “muddy-sediment,” reducing the contact time of the oily mass with the sludge at the end of the decantation is important. This can be performed to by pouring off the settled oil fraction into an empty container or eliminating the sludge through the discharge valve located on the conical bottom of the container.

## Figures and Tables

**Table 1:** Physicochemical characteristics of Coratina monocultivar EVOO during three months of decantation.

Racking Time (Days)	Solid Material Content (%, *w*/*v*)	∆ (%) ^1^	Water Content (%, *w*/*w*)	∆ (%)	Biophenols (mg TE Kg^−1^ oil) ^2^	∆ (%)
0 (Control)	0.140 ± 0.008 ^3,a^	0	0.210 ± 0.007 ^a^	0	773 ± 2 ^a^	0
30	0.065 ± 0.004 ^b^	54	0.152 ± 0.014 ^b^	28	758 ± 4 ^b^	2
60	0.041 ± 0.006 ^bc^	71	0.081 ± 0.005 ^c^	61	753 ± 3 ^b^	3
90	0.012 ± 0.007 ^c^	92	0.070 ± 0.008 ^c^	67	750 ± 6 ^b^	3

^1^ ∆ (%), % reduction at racking time compared to the control; ^2^, TE, tyrosol equivalent; ^3^, mean ± standard deviation (*n* = 3). Different letters in the same column indicate significant differences calculated using Duncan’s multiple range test (*p* < 0.05).

**Table 2:** Endogenous oleuropeinolytic activity of Coratina monocultivar EVOO evaluated during the decantation time and after seven months of storage.

Racking Time (Days)	β-Glucosidase (µg *p*-Nitrophenol mL^−1^ Oil)				Esterase (µg *p*-Nitrophenol mL^−1^ Oil)			
	Decantation Time	∆(%) ^1^	After Storage	∆ (%) ^2^	Decantation Time	∆ (%)	After Storage	∆ (%)
0 (Control)	0.20 ± 0.02 ^3,a^	0	0.20 ± 0.03 ^a^	0	1.85 ± 0.05 ^a^	0	0.18 ± 0.02 ^a^	0
30	0.19 ± 0.01 ^a^	5	0.17 ± 0.01 ^a^	15	1.65 ± 0.07 ^a^	11	0.15 ± 0.03 ^a^	17
60	0.11 ± 0.03 ^b^	45	0.09 ± 0.02 ^b^	55	0.92 ± 0.08 ^b^	50	0.13 ± 0.05 ^ab^	25
90	0.09 ± 0.01 ^b^	55	0.06 ± 0.00 ^b^	70	0.83 ± 0.07 ^b^	55	0.08 ± 0.01 ^b^	55

^1^ ∆ (%), % of enzymatic reduction at racking time compared to the control; ^2^ ∆ (%), % of enzymatic reduction at the end of storage compared to the control; ^3^ mean ± standard deviation (*n* = 3). Different letters in the same column indicate significant differences calculated using Duncan’s multiple-range test (*p* < 0.05).

**Table 3:** Microbiological analysis of Coratina monocultivar EVOO during three months of decantation and after seven months of storage.

Racking Time (Days)	Total Bacteria (Log CFU mL^−1^ Oil)		Total Yeasts (Log CFU mL^−1^ Oil)			Oleuropeinolytic Yeasts Evaluated during the Decantation Time	
	Decantation Time	After Storage	Decantation Time	∆ (%) ^1^	After Storage	β-Glucosidase^+^ Yeasts (%)	Esterase^+^ Yeasts (%)
0 (Control)	1.72 ± 0.28	0	2.48 ± 0.17 ^2,a^	0	1.94 ± 0.10	100 ± 0.90 ^ns^	64 ± 0.31 ^a^
30	0	0	1.83 ± 0.31 ^b^	26	0	95 ± 0.75 ^ns^	23 ± 0.09 ^b^
60	0	0	1.02 ± 0.14 ^c^	59	0	95 ± 0.57 ^ns^	10 ± 0.06 ^b^
90	0	0	0	100	0	0	0

^1^ ∆ (%), % of total yeast reduction at racking time compared to the control; ^2^ mean ± standard deviation (*n* = 3); different letters in the same column indicate significant differences calculated using Duncan’s multiple-range test (*p* < 0.05); ^ns^ not significant.

**Table 4:** Yeast species prevalence (%) in Coratina monocultivar EVOO during three months of decantation.

Yeast Species		Racking Time (Days)		
	0	30	60	90
	(Control)			
*Barnettozyma californica*	1 ^c^	3 ^c^	0	0
*Candida adriatica*	23 ^b^	77 ^a^	84 ^a^	0
*Kuraishia capsulata*	1 ^c^	0	0	0
*Nakazawaea molendinolei*	17 ^b^	20 ^b^	16 ^b^	0
*Yamadazyma terventina*	58 ^a^	0	0	0

Different letters in the same column indicate significant differences calculated using Duncan’s multiple-range test (*p* < 0.05).

**Table 5:** Prevalence (%) of five oil-borne yeast strains inoculated in Coratina monocultivar EVOO with different water contents after three months of incubation.

Yeast Strains	Source	Water Content (%)		
		0.07	0.21	0.30
*Barnettozyma californica* 2084	Leccino EVOO	0	0	0
*Candida adriatica* 2087	Coratina EVOO	85 ^a^	70 ^a^	72 ^a^
*Kuraishia capsulata* 2090	Coratina EVOO	0	0	5 ^b^
*Nakazawaea molendinolei* 2096	Coratina EVOO	10 ^b^	20 ^b^	8 ^b^
*Yamadazyma terventina* 2092	Don Carlo EVOO	5 ^b^	10 ^b^	15 ^b^

Different letters in the same column indicate significant differences calculated using Duncan’s multiple-range test (*p* < 0.05).

**Table 6:** Bitterness parameters of Coratina monocultivar EVOO evaluated during the decantation time and after seven months of storage.

Racking Time (Days)	Bitterness Parameters											
	During Decantation						After Storage					
	R-Index ^1^	∆ (%) ^2^	Sensory Attribute	∆ (%) ^2^	K_225_^3^	∆ (%) ^2^	R-Index	∆ (%) ^4^	Sensory Attribute	∆ (%) ^4^	K_225_	∆ (%) ^4^
0 (Control)	0.16 ^a^	0	4.8 ± 0.1 ^5,a^	0	0.757 ± 0.001 ^a^	0	0.14 ^a^	13	4.0 ± 0.2 ^a^	17	0.675 ± 0.002 ^a^	11
30	0.07 ^ab^	56	4.6 ± 0.2 ^a^	4	0.726 ± 0.002 ^ab^	4	0.06 ^ab^	14	3.8 ± 0.3 ^ab^	17	0.668 ± 0.001 ^a^	8
60	0.04 ^b^	75	3.8 ± 0.3 ^b^	21	0.715 ± 0.004 ^b^	6	0.04 ^b^	0	3.6 ± 0.1 ^b^	5	0.644 ± 0.005 ^b^	10
90	0.04 ^b^	75	3.2 ± 0.1 ^c^	33	0.668 ± 0.003 ^c^	12	0.04 ^b^	0	3.0 ± 0.4 ^c^	6	0.626 ± 0.003 ^b^	6

^1^ R-index ((free tyrosol + free hydroxytyrosol)/(free tyrosol + free hydroxytyrosol + secoiridoid derivatives)); ^2^ ∆ (%), % reduction during decantation compared to the control; ^3^ K_225_, bitterness index; ^4^ ∆ (%), % reduction at the end of storage compared to the corresponding racking time; ^5^, median of the values; EVOO, extra virgin olive oil. Different letters in the same column indicate significant differences calculated using Duncan’s multiple-range test (*p* < 0.05).

**Table 7:** Sensory profile of Coratina monocultivar EVOO evaluated at racking time and after seven months of storage.

Racking Time (Days)	Fruitiness				Pungency				Defects	Merceological Class	
	At Racking Time	∆ (%) ^1^	After Storage	∆ (%) ^2^	At Racking Time	∆ (%) ^1^	After Storage	∆ (%) ^2^		At Racking Time	After Storage
0 (Control)	5.0 ± 0.3 ^3,a^	0	4.0 ± 0.2 ^a^	20	4.0 ± 0.2 ^ns^	0	3.8 ± 0.1 ^ns^	5	0	EVOO	EVOO
30	4.5 ± 0.2 ^ab^	10	3.8 ± 0.3 ^ab^	16	4.0 ± 0.3 ^ns^	0	3.6 ± 0.2 ^ns^	10	0	EVOO	EVOO
60	3.8 ± 0.1 ^b^	24	3.7 ± 0.2 ^b^	3	3.8 ± 0.1 ^ns^	5	3.6 ± 0.3 ^ns^	5	0	EVOO	EVOO
90	3.8 ± 0.2 ^b^	24	3.7 ± 0.1 ^b^	3	3.7 ± 0.2 ^ns^	8	3.6 ± 0.1 ^ns^	3	0	EVOO	EVOO

^1^ ∆ (%), % of reduction during the decantation compared to the control; ^2^ ∆ (%), % reduction at the end of storage compared to the racking time; ^3^, median values; EVOO, extra virgin olive oil; different letters in the same column indicate significant differences calculated using Duncan’s multiple-range test (*p* < 0.05); ns, not significant.

**Table 8:** Analytical indices of Coratina monocultivar EVOO evaluated at racking time and at the end of storage.

Racking Time (Days)	Free Fatty Acid (% Oleic Acid)		Peroxide Value (meq O_2_ kg^−1^)		K_232_		K_270_		∆K	
	At Racking Time	After Storage	At Racking Time	After Storage	At Racking Time	After Storage	At Racking Time	After Storage	At Racking Time	After Storage
0	0.28 ± 0.01^1 ns^	0.28 ± 0.04 ^ns^	4.70 ± 0.28 ^b^	4.95 ± 0.92 ^b^	1.808 ± 0.004 ^b^	2.060 ± 0.002 ^ns^	0.157 ± 0.001 ^ns^	0.176 ± 0.005 ^ns^	−0.003	−0.003
30	0.29 ± 0.02 ^ns^	0.25 ± 0.01 ^ns^	4.05 ± 0.21 ^b^	5.80 ± 0.17 ^b^	1.928 ± 0.002 ^b^	1.994 ± 0.007 ^ns^	0.155 ± 0.005 ^ns^	0.155 ± 0.006 ^ns^	−0.002	−0.002
60	0.31 ± 0.01 ^ns^	0.26 ± 0.01 ^ns^	6.05 ± 0.19 ^a^	6.80 ± 0.11 ^a^	2.041 ± 0.005 ^a^	2.120 ± 0.004 ^ns^	0.148 ± 0.002 ^ns^	0.160 ± 0.001 ^ns^	−0.003	−0.003
90	0.28 ± 0.05 ^ns^	0.24 ± 0.05 ^ns^	6.55 ± 0.49 ^a^	8.00 ± 0.14 ^a^	2.070 ± 0.010 ^a^	2.317 ± 0.001 ^ns^	0.145 ± 0.001 ^ns^	0.158 ± 0.004 ^ns^	−0.003	−0.003
Limit for EVOO class										
	≤0.80		≤20		≤2.50		≤0.22		≤0.010	

^1^, mean ± standard deviation (*n* = 3); EVOO, extra virgin olive oil; Different letters in the same column indicate significant differences calculated using Duncan’s multiple- range test (*p* < 0.05); ^ns^, not significant.

## Data Availability

Not applicable.
